# Correction: Structural variation underlies functional diversity at methyl salicylate loci in tomato

**DOI:** 10.1371/journal.pgen.1011125

**Published:** 2024-01-18

**Authors:** Manoj Sapkota, Lara Pereira, Yanbing Wang, Lei Zhang, Yasin Topcu, Denise Tieman, Esther van der Knaap

An additional affiliation is missing for the fifth author. Yasin Topcu is also affiliated with Batı Akdeniz Agricultural Research Institute, 07100 Antalya, Turkey
