# Supplementary figures and images for: Structural variation underlies functional diversity at methyl salicylate loci in tomato
Source: PLoS Genet. 2023 May 4;19(5):e1010751. doi: 10.1371/journal.pgen.1010751 (PMC10187894; doi:10.1371/journal.pgen.1010751)

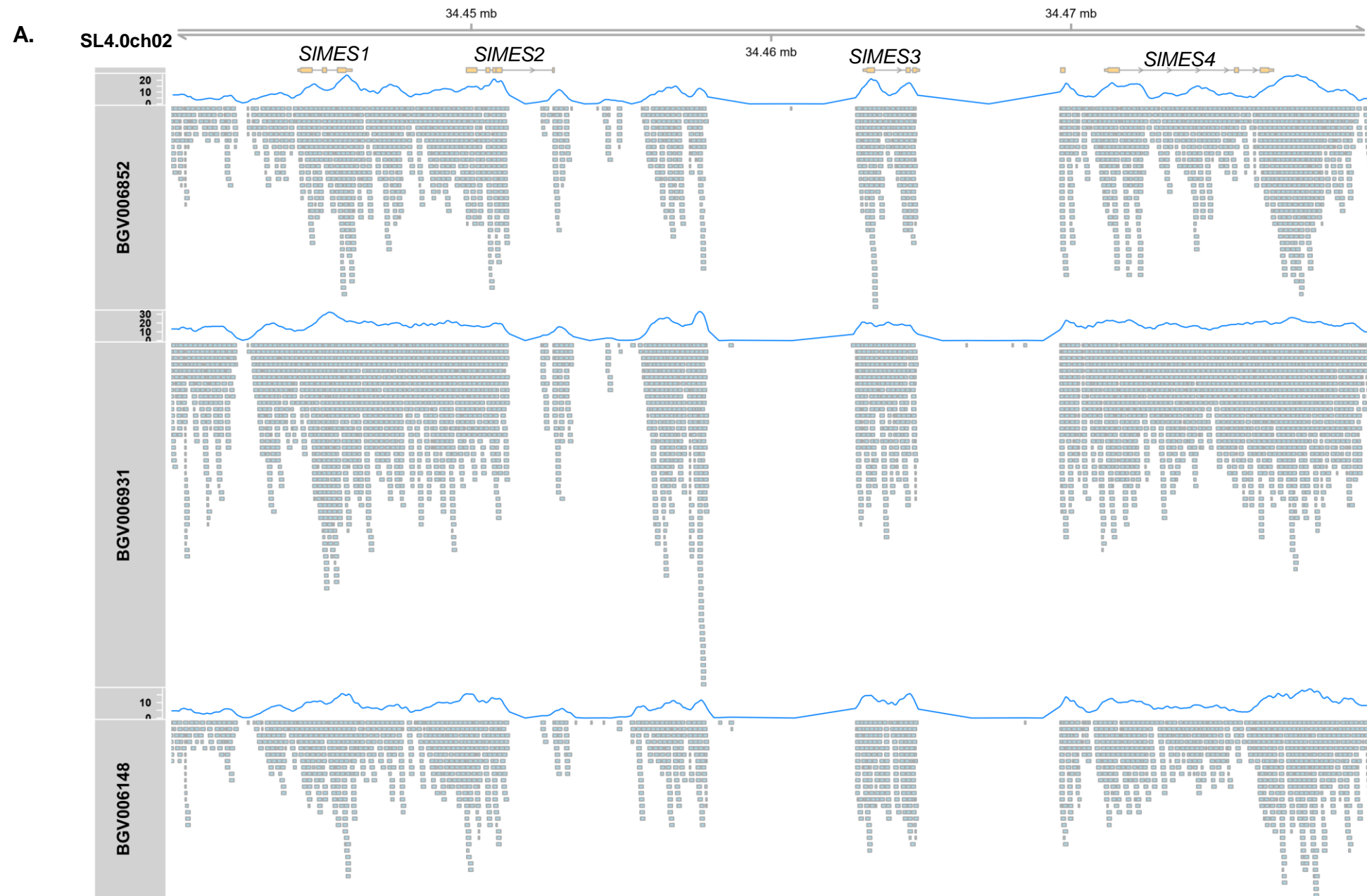

B.

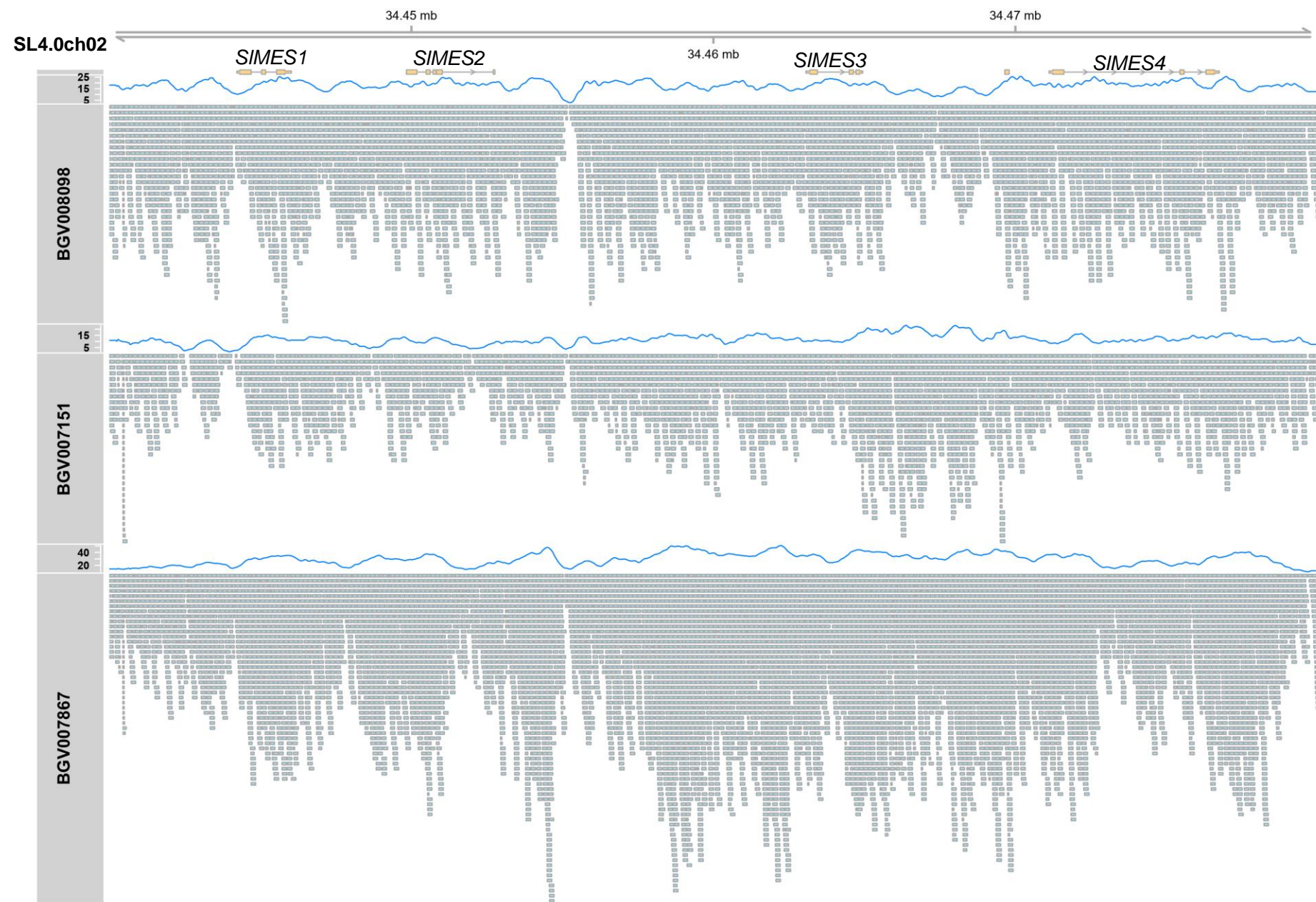

Supplement: S1 Fig — (A) Alignment of three highest methyl salicylate producing accessions against the SL4.0 genome build at the MES locus. (B) Alignment of three least methyl salicylate producing accessions against the SL4.0 genome build at the MES locus. Yellow boxes represent gene models. (PDF) [file pgen.1010751.s001.pdf]

SL4.0ch02

34.44 mb

34.45 mb

34.46 mb

34.47 mb

34.48 mb

Rio Grande

6  
4  
2  
0

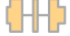  
*SIMES1*

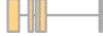  
*SIMES2*

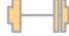  
*SIMES3*

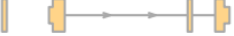  
*SIMES4*

RioGrande

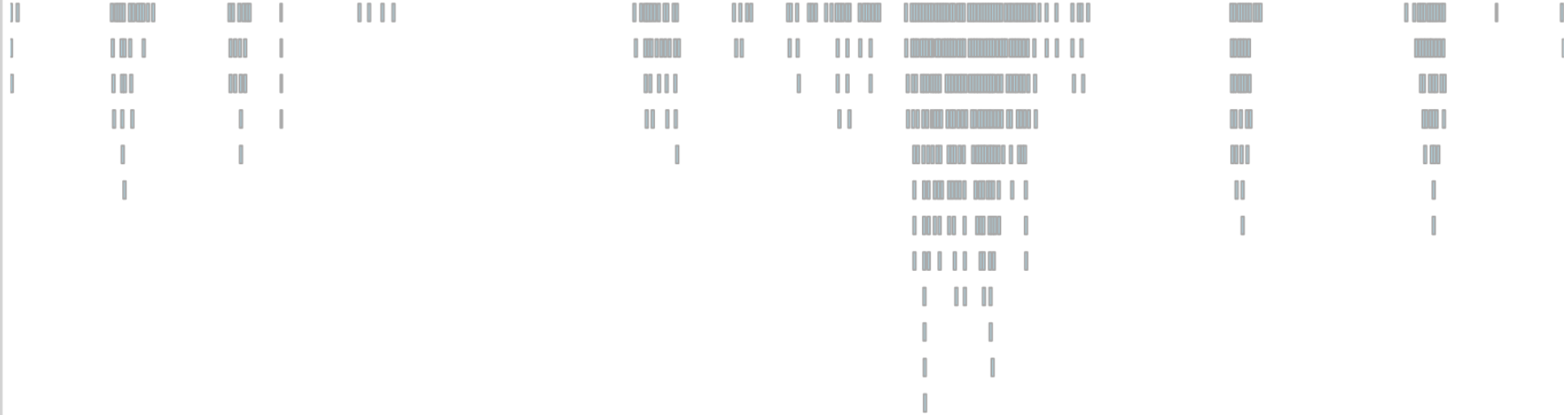

Supplement: S4 Fig — Alignment for SlMES1, SlMES2, SlMES3 and SlMES4. Yellow boxes represent gene models. (PDF) [file pgen.1010751.s004.pdf]

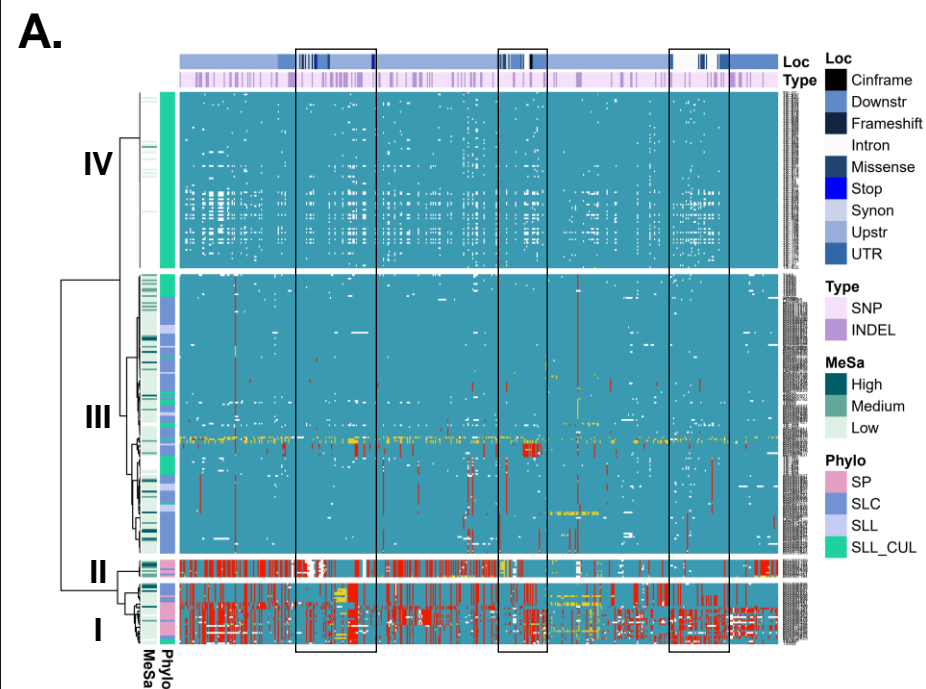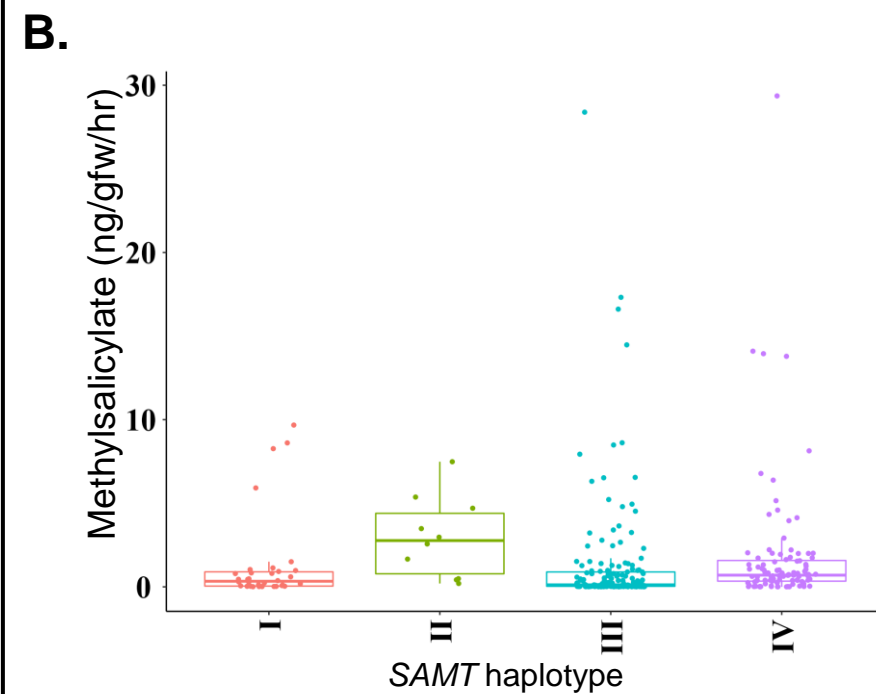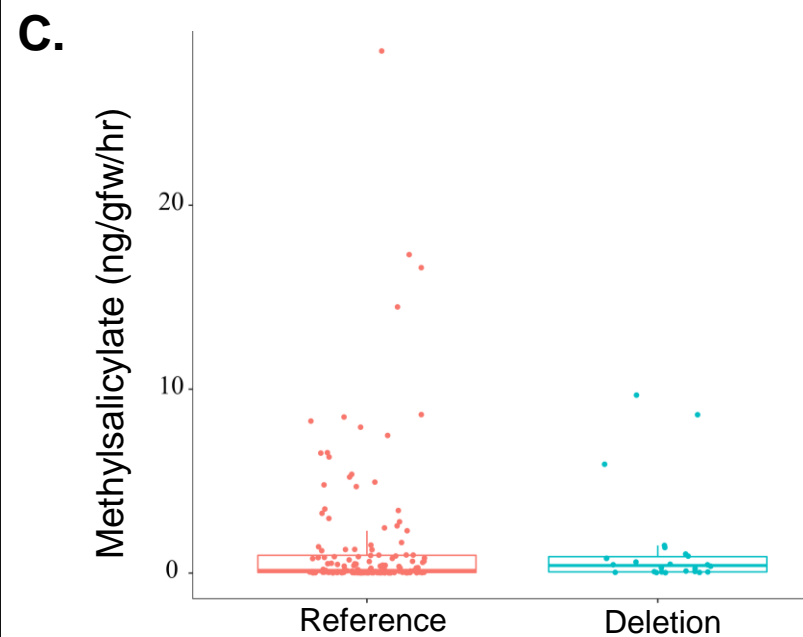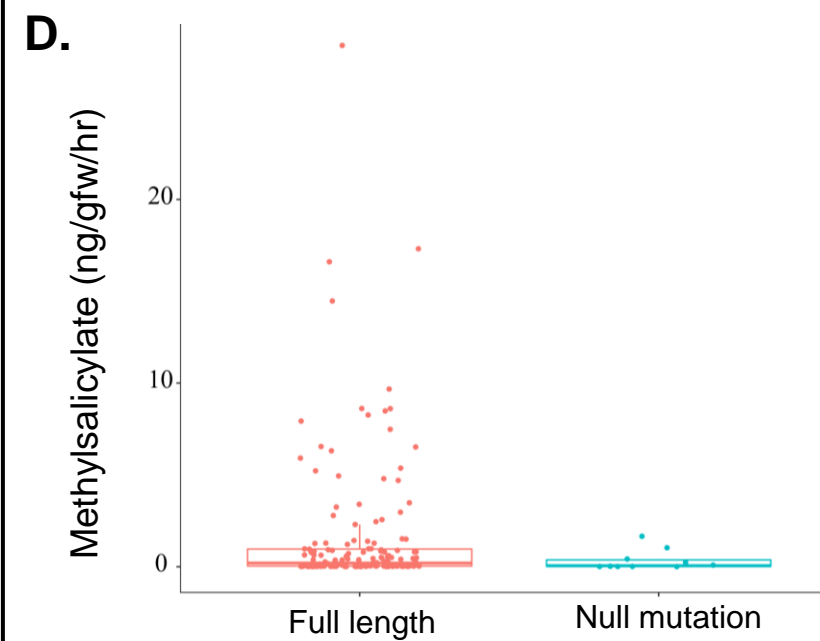

Supplement: S5 Fig — (A) Heatmap representing the genotypes of accessions (rows) for the polymorphisms identified (columns). Reference genotypes are represented in blue, alternate in red, heterozygous in yellow and missing data in white. The black rectangular box represents the position of the genes in the locus. (B) Distribution of methyl salicylate in red fruits in different accessions among different SAMT1 haplotypes. (C) Distribution of methyl salicylate in red fruits in different accessions with and without deletion in the SAMT1 locus. (D) Distribution of methyl salicylate between accessions with and without null mutations of Solyc09g091530. (PDF) [file pgen.1010751.s005.pdf]

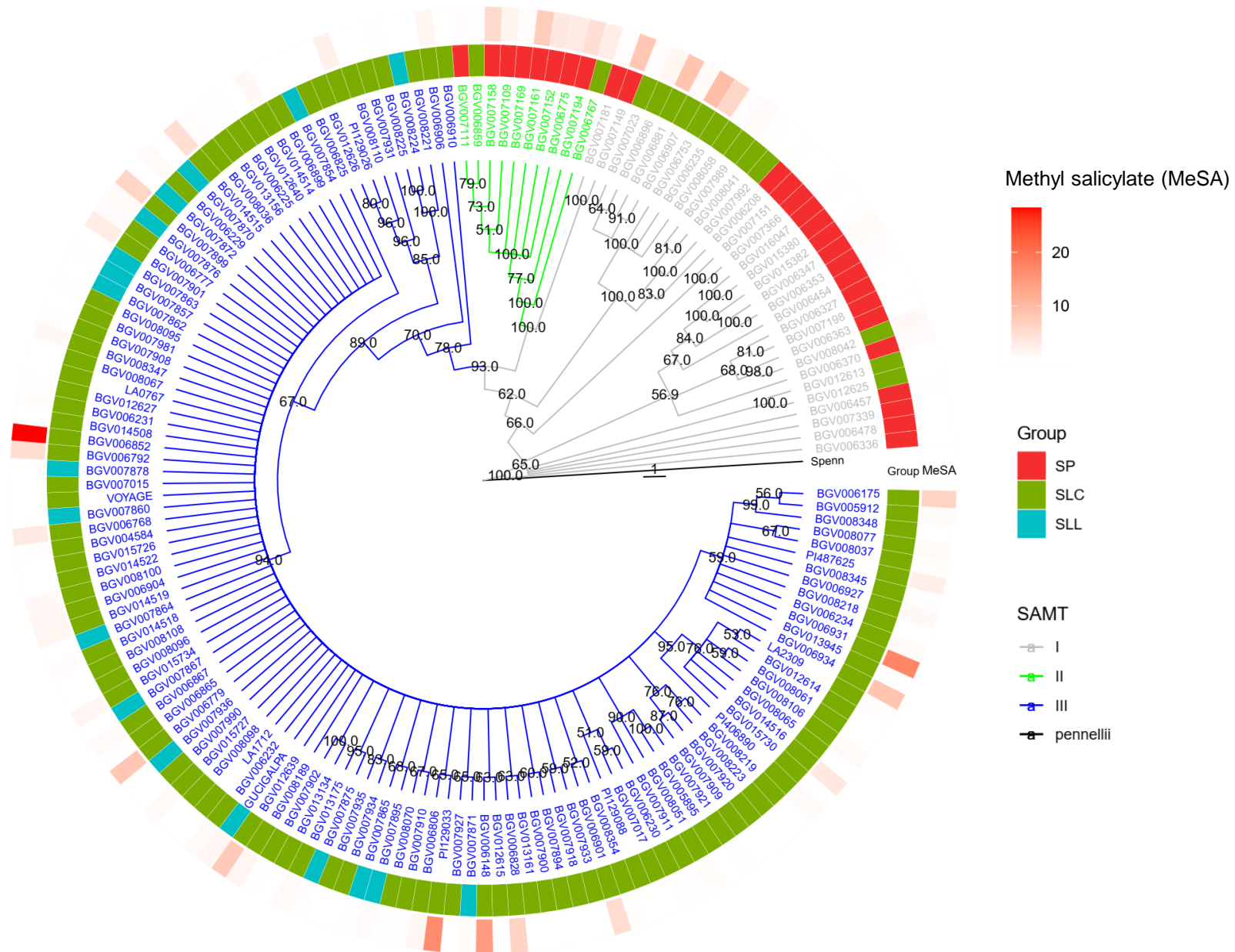

Supplement: S6 Fig — Different colors of accessions and branches represent different SAMT1 haplotypes. The outer two concentric circles represent the grouping of accessions (SP, SLC and SLL) and corresponding methyl salicylate levels (MeSA) in the accessions respectively. The numbers on the branches represent the bootstrap values. (PDF) [file pgen.1010751.s006.pdf]

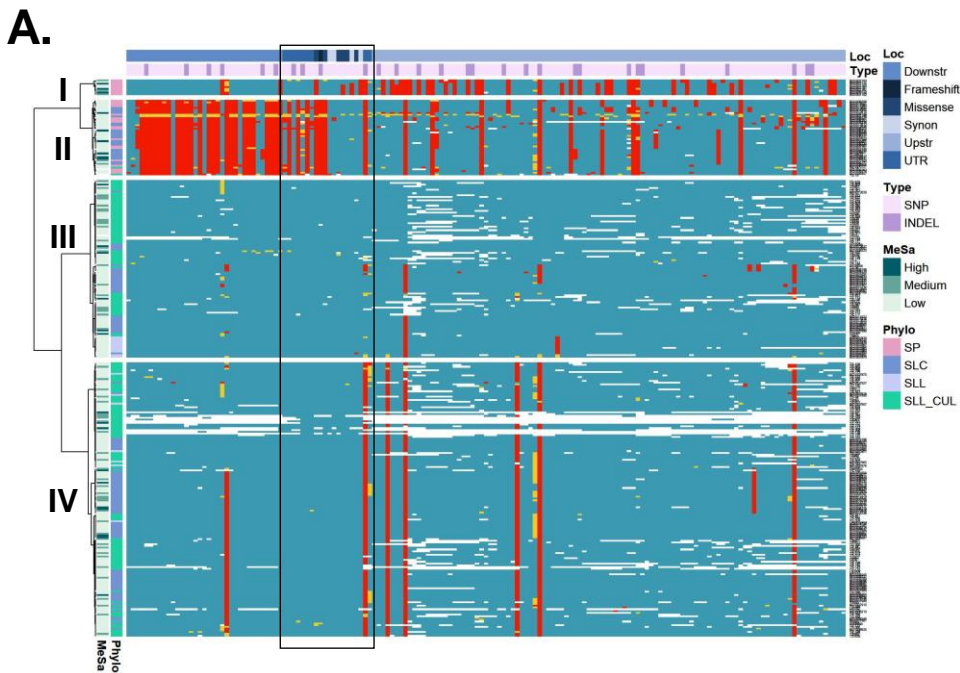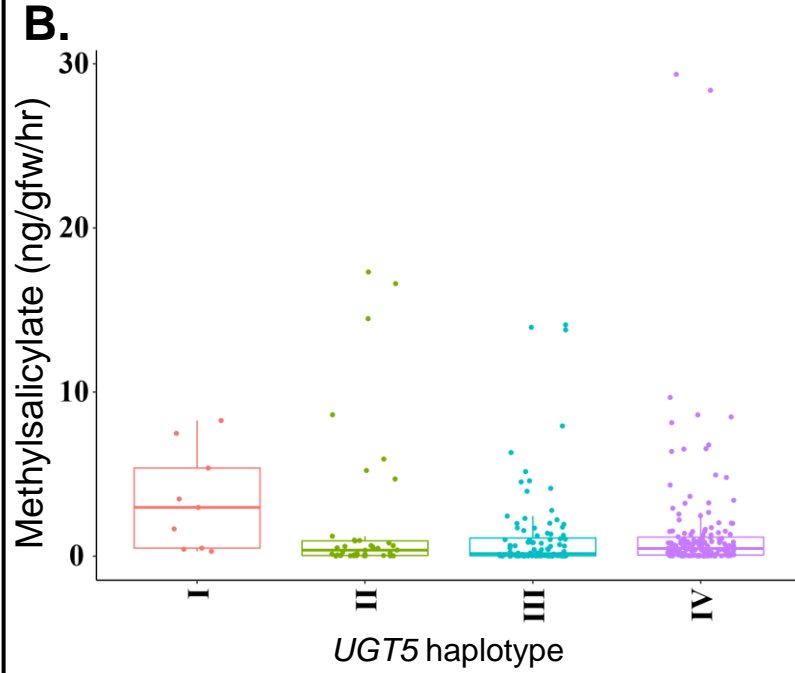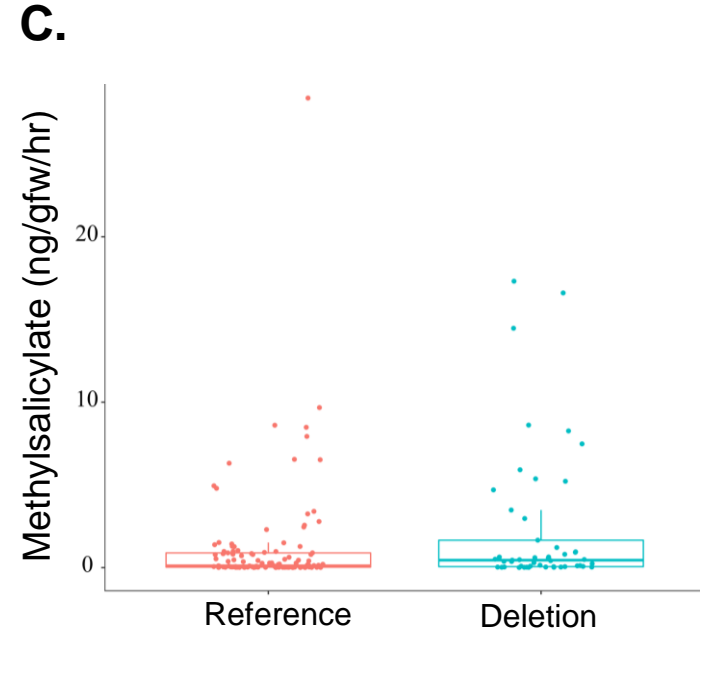

Supplement: S7 Fig — (A) Heatmap representing the genotypes of accessions (rows) for the polymorphisms identified (columns). Reference genotypes are represented in blue, alternate in red, heterozygous in yellow and missing data in white. The black rectangular box represents the position of the gene in the locus. (B) Distribution of methyl salicylate in red fruits in different accessions among different UGT5 haplotypes. (C) Distribution of methyl salicylate in red fruits in different accessions with and without deletion in the promoter of SlUGT5. (PDF) [file pgen.1010751.s007.pdf]

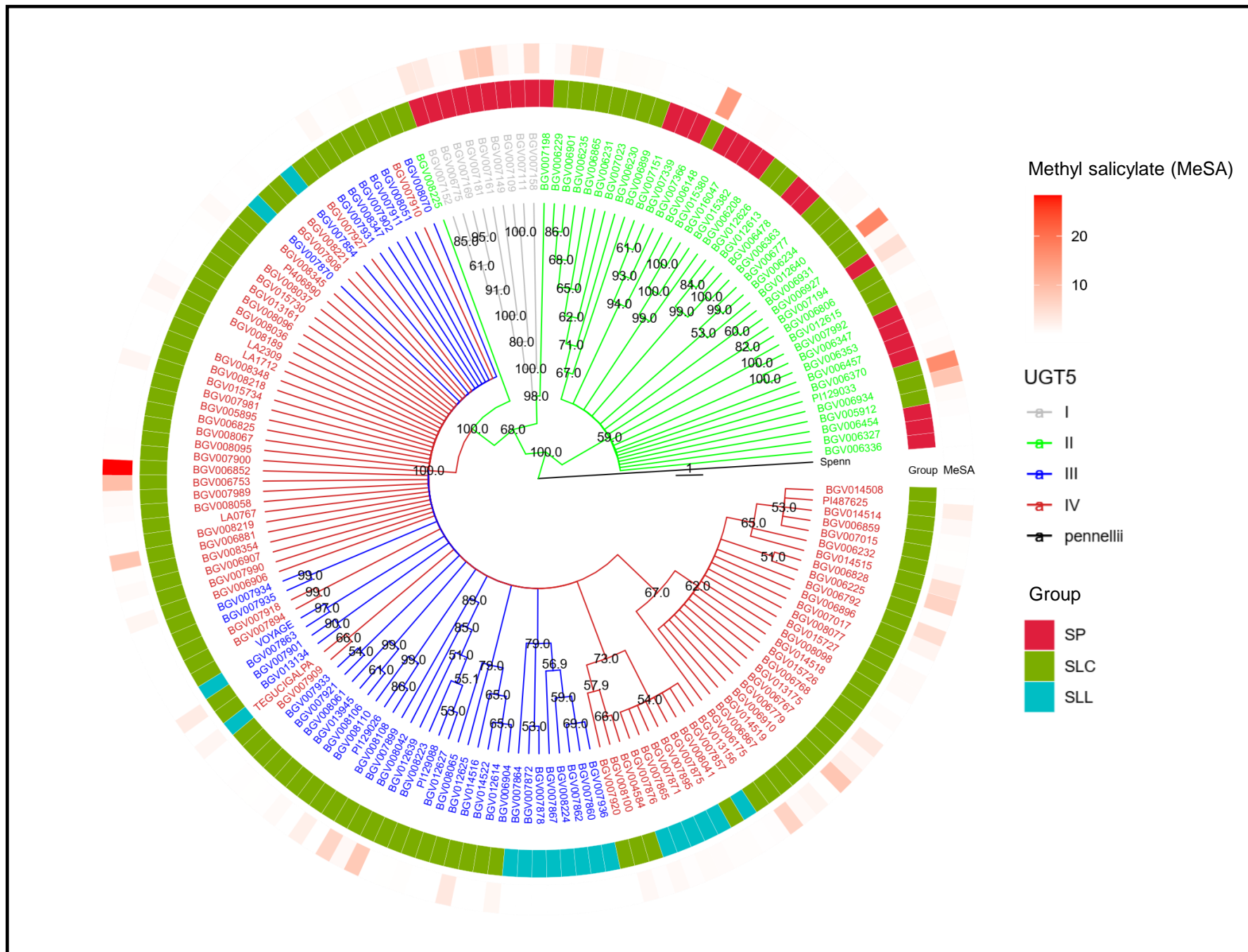

Supplement: S8 Fig — Different colors of accessions and branches represent different UGT5 haplotypes. The outer two concentric circles represent the grouping of accessions (SP, SLC and SLL) and corresponding methyl salicylate levels (MeSA) in the accessions respectively. The numbers on the branches represent the bootstrap values. (PDF) [file pgen.1010751.s008.pdf]
