# Supplementary material for: Structural variation underlies functional diversity at methyl salicylate loci in tomato
Source: PLoS Genet. 2023 May 4;19(5):e1010751. doi: 10.1371/journal.pgen.1010751 (PMC10187894; doi:10.1371/journal.pgen.1010751)
Supplement: S2 Fig — Alignments of (A) SlMES1 (B) SlMES2 (C) SlMES3 and (D) SlMES4 (PDF) [file pgen.1010751.s002.pdf]

A.

```
MES1hap5     MEANKKQGKHFVLVHGAGHGAWCWYKLKPLLEAAGHKVTALDLAASGIDLRKIEQLHTLH     60
MES1hap8     MEANKKQGKHFVLVHGAGHGAWCWYKLKPLLEAAGHKVTALDLAASGIDLRKIEQLHTLH     60
MES1hap1     MEANKKQGKHFVLVHGAGHGAWCWYKLKPLLEAAGHKVTALDLAASGIDLRKIEQLHTLH     60
MES1hap3     MEANKKQGKHFVLVHGAGHGAWCWYKLKPLLEAAGHKVTALDLAASGIDLRKIEQLHTLH     60
MES1hap2     MEANKKQGKHFVLVHGAGHGAWCWYKLKPLLEAAGHKVTALDLAASGIDLRKIEQLHTLH     60
MES1hap4     MEANKKQGKHFVLVHGAGHGAWCWYKLKPLLEAAGHKVTALDLAASGIDLRKIEQLHTLH     60
MES1hap6     MEANKKQGKHFVLVHGAGHGAWCWYKLKPLLEAAGHKVTALDLAASGIDLRKIEQLHTLH     60
MES1hap7     MEANKKQGKHFVLVHGAGHGAWCWYKLKPLLEAAGHKVTALDLAASGIDLRKIEQLHTLH     60
             ************************************************************

MES1hap5     DYTLPLLELMESLPQEEKVILVGHSLGGMNLALAMEKYPKKIYAAVFLAAFMPDSIHISS     120
MES1hap8     DYTLPLLELMESLPQEEKVILVGHSLGGMNLALAMEKYPKKIYAAVFLAAFMPDSIHISS     120
MES1hap1     DYTLPLLELMESLPQEEKVILVGHSLGGMNLALAMEKYPKKIYAAVFLAAFMPDSIHISS     120
MES1hap3     DYTLPLLELMESLPQEEKVILVGHSLGGMNLALAMEKYPKKIYAAVFLAAFMPDSIHISS     120
MES1hap2     DYTLPLLELMESLPQEEKVILVGHSLGGMNLALAMEKYPKKIYAAVFLAAFMPDSIHISS     120
MES1hap4     DYTLPLLELMESLPQEEKVILVGHSLGGMNLALAMEKYPKKIYAAVFLAAFMPDSIHISS     120
MES1hap6     DYTLPLLELMESLPQEEKAILVGHSLGGMNLALAMEKYPKKIYAAVFLAAFMPDSIHISS     120
MES1hap7     DYTLPLLELMESLPQEEKAILVGHSLGGMNLALAMEKYPKKIYAAVFLAAFMPDSIHISS     120
             ******************.*****************************************

MES1hap5     YVMDQNFNGKFLKYNERTPAENWLDTQFLPYGTPEEPLTSMTFGPKFLADKLYQLSPPED     180
MES1hap8     YVMDQNFNGKFLKYNERTPAENWLDTQFLPYGTPEEPLTSMTFGPKFLADKLYQLSPPED     180
MES1hap1     YVMDQ--------YNERTPAENWLDTQFLPYGTPEEPLTSMTFGPKFLADKLYQLSPPED     172
MES1hap3     YVMDQ--------YNERTPAENWLDTQFLPYGTPEEPLTSMTFGPKFLADKLYQLSPPED     172
MES1hap2     YVMDQ--------YNERTPAENWLDTQFLPYGTPEEPLTSMTFGPKFLADKLYRLSPPED     172
MES1hap4     YVMDQ--------YNERTPAENWLDTQFLPYGTPEEPLTSMTFGPKFLADKLYRLSPPED     172
MES1hap6     YVMDQ--------YNERTPAENWLDTQFLPYGTPEEPLTSMTFGPKFLADKLYRLSPPED     172
MES1hap7     YVMDQ--------YNERTPAENWLDTQFLPYGTPEEPLTSMTFGPKFLADKLYRLSPPED     172
             *****        ****************************************:******

MES1hap5     VALGLSLVRTSSLFLEDLSKAKYLTDEGYGSVKRVYVVCTEDKGISKEFQQWQIDNIGVT     240
MES1hap8     VALGLSLVRTSSLFLEDLSKAKYLTDEGYGSVKRVYVVCTEDKGISKEFQQWQIDNIGVT     240
MES1hap1     VALGLSLVRTSSLFLEDLSKAKYLTDEGYGSVKRVYVVCTEDKGISKEFQQWQIDNIGVT     232
MES1hap3     VALGLSLVRTSSLFLEDLSKAKYLTDEGYGSVKRVYVVCTEDKGISKEFQQWQIDNIGVT     232
MES1hap2     VALGLSLVRTSSLFLEDLSKAKYLTDEGYGSVKRVYVVCTEDKGIAKEFQQWQIDNIGVT     232
MES1hap4     VALGLSLVRTSSLFLEDLSKAKYLTDEGYGSVKRVYVVCTEDKGIAKEFQQWQIDNIGVT     232
MES1hap6     VALGLSLVRTSSLFLEDLSKAKYLTDEGYGSVKRVYVVCTEDKGISKEFQQWQIDNIGVT     232
MES1hap7     VALGLSLVRTSSLFLEDLSKAKYLTDEGYGSVKRVYVVCTEDKGISKEFQQWQIDNIGVT     232
             *********************************************:**************

MES1hap5     EAKEIKGADHMAMLCMPKKLCDTLVEIADKYN   272
MES1hap8     EAKEIKGADHMAMLCMPKKLCDTLVEIADKYN   272
MES1hap1     EAKEIKGADHMAMLCMPKKLCDTLVEIADKYN   264
MES1hap3     EAKEIKGADHMAMLCMPKKLCDTLVEIADKYN   264
MES1hap2     EAKEIKGADHMAMLCMPKKLCDTLVEIADKYN   264
MES1hap4     EAKEIKGADHMAMLCMPKKLCDTLVEIADKYN   264
MES1hap6     EAKEIKGADHMAMLCMPKKLCDTLVEIADKYN   264
MES1hap7     EAKEIKGADHMAMLCMPKKLCDTLVEIADKYN   264
             ********************************
```

B.

```
MES2hap5     MEGMKKHFVLVH----GGWCWYKLKPLLEAAGHKVTTFDLAASGIDLRKIEQLHTLHDYT     56
MES2hap6     MEGMKKHFVLVH----GGWCWYKLKPLLEAAGHKVTTFDLAASGIDLRKIEQLHTLHDYT     56
MES2hap7     MEGMKKHFVLVH----GGWCWYKLKPLLEAAGHKVTTFDLAASGIDLRKIEQLHTLHDYT     56
MES2hap8     MEGMKKHFVLVH----GGWCWYKLKPLLEAAGHKVTTFDLAASGIDLRKIEQLHTLHDYT     56
MES2hap1     MEGMKKHFVLVHGACHGGWCWYKLKPLLEAAGHKVTTFDLAASGIDLRKIEQLHTLHDYT     60
MES2hap2     MEGMKKHFVLVH----GGWCWYKLKPLLEAAGHKVTTFDLAASGIDLRKIEQLHTLHDYT     56
MES2hap3     MEGMKKHFVLVH----GGWCWYKLKPLLEAAGHKVTTFDLAASGIDLRKIEQLHTLHDYT     56
MES2hap4     MEGMKKHFVLVH----GGWCWYKLKPLLEAAGHKVTTFDLAASGIDLRKIEQLHTLHDYT     56
             ************    ********************************************

MES2hap5     LPLFKLMESLPQEEKVILVGHSFGGMNLGLVMEKYPEKIYVAVFLAAFMPDSNHISSYVL     116
MES2hap6     LPLFKLMESLPQEEKVILVGHSFGGMNLGLVMEKYPEKIYVAVFLAAFMPDSNHISSYVL     116
MES2hap7     LPLFKLMESLPQEEKVILVGHSFGGMNLGLVMEKYPEKIYVAVFLAAFMPDSNHISSYVL     116
MES2hap8     LPLFKLMESLPQEEKVILVGHSFGGMNLGLVMEKYPEKIYVAVFLAAFMPDSNHISSYVL     116
MES2hap1     LPLFKLMESLPQEEKVILVGHSFGGMNLGLVMEKYPEKIYVAVFLAAFMPDSNHISSYVL     120
MES2hap2     LPLFKLMESLPQEEKVILVGHSFGGMNLGLVMEKYPEKIYVAVFLAAFMPDSNHISSYVL     116
MES2hap3     LPLFKLMESLPQEEKVILVGHSFG------------------------------------     80
MES2hap4     LPLFKLMESLPQEEKVILVGHSFG------------------------------------     80
             ************************

MES2hap5     D------EYYEQTPEEKWLDTQFLPYGSPEEQLTSMTFGPKYLANKLYQLCSPEDVALGL     170
MES2hap6     D------EYYEQTPEEKWLDTQFLPYGSPEEQLTSMTFGP-YLANKLYQLCSPEDVALGL     169
MES2hap7     D------EYYEQTPEEKWLDTQFLPYGSPEEQLTSMTFGP-YLANKLYQLCSPEDVALGL     169
MES2hap8     D------EYYEQTPEEKWLDTQFLPYGSPEEQLTSMTFGP-YLANKLYQLCSPEDVALGL     169
MES2hap1     D------EYYEQTPEEKWLDTQFLPYGSPEEQLTSMTFGPKYLAYKLYQLCSPEDVALGL     174
MES2hap2     DNIPNTLSYYEQTPEEKWLDTQFLPYGSPEEQLTSMTFGPKYLANKLYQLCSPEDVALGL     176
MES2hap3     --------------EEKWLDTQFLPYGSPEEQLTSMTFGPKYLAYKLYQLCSPEDVALGL     126
MES2hap4     --------------EEKWLDTQFLPYGSPEEQLTSMTFGPKYLANKLYQLCSPEDVALGL     126
                           ************************** *** ***************

MES2hap5     SLVRSSSLFRKDLSKAKYLTDEGYGSVKRVYIMCSEDKGMPKEFGRWQIDNIGVTEEKEI     230
MES2hap6     SLVRSSSLFRKDLSKAKYLTDEGYGSVN--------------------------------     197
MES2hap7     SLVRSSSLFRKDLSKAKYLTDEGYGSVKRVYIMCSEDKGMPKEFGRWQIDNIGVTEEKEI     229
MES2hap8     SLVRSSSLFRKDLSKAKYLTDEGYGSVKRVYIMCSEDKGMPKEFGRWQIDNIGVTEEKEI     229
MES2hap1     SLVRSSSLFRKDLSKAKYLTDEGYGSVKRVYIMCSEDKGIPKEFGRWLIDNIGVTEEKEI     234
MES2hap2     SLVRSSSLFRKDLSKAKYLTDEGYGSVKRVYIMCSEDKGIPKEFGRWLIDNIGVTEEKEI     236
MES2hap3     SLVRSSSLFRKDLSKAKYLTDEGYGSVKRVYIMCSEDKGIPKEFGRWLIDNIGVTEEKEI     186
MES2hap4     SLVRSSSLFRKDLSKAKYLTDEGYGSVKRVYIMCSEDKGIPKEFGRWLIDNIGVTEEKEI     186
             ***************************:

MES2hap5     KGADHMAMLSMPKELCDILFEIAHKYNNPSSTHLNNYLLLLSNRK     275
MES2hap6     ---------------------------------------------     197
MES2hap7     KGADHMAMLSMPKELCDILFEIAHKYNNPSSTHLNNYLLLLSNRK     274
MES2hap8     KGADHMAMLSMPKELCDILFEIAHKYNNPSSTHLNNYLLLLSNRK     274
MES2hap1     KGADHMAMLSMPKELCDILFEIANKYNSPSSTHLNNYLLLLSTRK     279
MES2hap2     KGADHMAMLSMPKELCDILFEIANKYNSPSSTHLNNYLLLLSTRK     281
MES2hap3     KGADHMAMLSMPKELCDILFEIANKYNSPSSTHLNNYLLLPSTRK     231
MES2hap4     KGADHMAMLSMPKELCDILFEIANKYNSPSSTHLNNYLLLLSTRK     231
```

C.

```
MES3hap1        MEVMKKHFVLVHGACHGSWCWYKLKPLLEAAGHKVTALDMAASGIDLRKIEEIRSLVDYT      60
MES3hap3        MEVMKKHFVLVHGACHGSWCWYKLKPLLEAAGHKVTALDMAASGIDLRKIEEIRSLVDYT      60
MES3hap2        MEVMKKHFVLVHGACHGSWCWYKLKPLLEAAGHKVTALDMAASGIDLRKIEEIRSLVDYT      60
MES3hap4        MEVMKKHFVLVHGACHGSWCWYKLKPLLEAAGHKVTALDMAASGIDLRKIEEIRSLVDYT      60
MES3hap5        ---------------------------------------MAASGIDLRKIEEIRTLVDYT      21
MES3hap6        ---------------------------------------MAASGIDLRKIEEIRTLVDYT      21
MES3hap7        ---------------------------------------MAASGIDLRKIEEIRTLVDYT      21
                                                       ***************:*****

MES3hap1        APLMEFMESLPHEEKVVLVGHSLGGMNLALAMEKYPKKIYAAVFLAALMPDSAHMSSYVL      120
MES3hap3        APLMEFMESLPHEEKVVLVGHSLGGMNLALAMEKYPKKIYAAVFLAALMPDSAHMSSYVL      120
MES3hap2        APLMEFMESLPHEEKVVLVGHSFGGMNLALAMEKYPKKIYAAVFLAALMPDSAHMSSYVL      120
MES3hap4        APLMEFMESLPHEEKVVLVGHSFGGMNLALAMEKYPKKIYAAVFLAALMPDSAHMSSYVL      120
MES3hap5        APLMEFMESLPHEEKVVLVGHSLGGMNLALAMEKYPKKIYAAVFLAALMPDSAHMSSYVL      81
MES3hap6        APLMEFMESLPHEEKVVLVGHSLGGMNLALAMEKYPKKIYAAVFLAALMPDSAHMSSYVL      81
MES3hap7        APLMEFMESLPHEEKVVLVGHSLGGMNLALAMEKYPKKIYAAVFLAALMPDSAHMSSYVL      81
                **********************:*************************************

MES3hap1        DQY----------------LERTPTKNWCDTQFVSYGSPEEPLTSIILDPKLLAHRFYQL      164
MES3hap3        DQY----------------LERTPTKNWCDTQFVSYGSPEEPLTSIILDPKLLAHRFYQL      164
MES3hap2        DQY----------------LERTPTKNWCDTQFVSYGSPEEPLTSIILDPKLLAHRFYQL      164
MES3hap4        DQY----------------LERTPTKNWCDTQFVSYGSPEEPLTSIILDPKLLAHRFYQL      164
MES3hap5        DQLAMCVNLVAEPGFSRKQFERTPTKKWCDTQFVSYGSPEEPLTSIILDPKLLAHRFYQL      141
MES3hap6        DQLAMCVNLVAEPGFSRKQFERTPTKNWCDTQFVSYGSPEEPLTSIILDPKLLAHRFYQL      141
MES3hap7        DQLAMCVNLVAEPGFSRKQFERTPTKNWCDTQFVSYGSPEEPLTSIILDPKLLAHRFYQL      141
                **                 :******:*********************************

MES3hap1        CSSEDVALASTLIRPSSLFIEDLSKAKYFTDEGYGSVNKVYIIYIICTEDKALPKEFQQW      224
MES3hap3        CSSE--------------------------------------------------------      168
MES3hap2        CSSE--------------------------------------------------------      168
MES3hap4        CSSE--------------------------------------------------------      168
MES3hap5        CSPEDVALASTLIRPSSLFIEDLSKAKYFTDEGYGSVNKVYIMLE---------------      186
MES3hap6        CSPE--------------------------------------------------------      145
MES3hap7        CSPE--------------------------------------------------------      145
                ** *

MES3hap1        QIDNIESVTDVKEMKGADHMAMLCMPKQLCDTLLEIVQKHN 265
MES3hap3        ----------------------------------------- 168
MES3hap2        ----------------------------------------- 168
MES3hap4        ----------------------------------------- 168
MES3hap5        ----------------------------------------- 186
MES3hap6        ----------------------------------------- 145
MES3hap7        ----------------------------------------- 145
```

D.

```
MES4hap1        MEPIKKQGRHFVLVHGACHGGWCWYKLKPLLEVAGHKVTTLDLAASGIDLIKIEQLHTFH      60
MES4hap3        MEPIKKQGRHFVLVHGACHGGWCWYKLKPLLEVAGHKVTTLDLAASGIDLIKIEQLHTFH      60
MES4hap8        ------------------------------------------------------------      0
MES4hap2        MEPIKKQGRHFVLVHGACHGGWCWYKLKPLLEVAGHKVTTLDLAASGIDLRKIEQLHTFH      60
MES4hap4        MEPIKKQGRHFVLVHGACHGGWCWYKLKPLLEVAGHKVTTLDLAASGIDLRKIEQLHTFH      60
MES4hap5        MEPIKKQGRHFVLVHGACHGGWCWYKLKPLLEVAGHKVTTLDLAASGIDLRKIEQLHTFH      60
MES4hap6        MEPIKKQGRHFVLVHGACHGGWCWYKLKPLLEVAGHKVTTLDLAASGIDLRKIEQLHTFH      60
MES4hap7        MEPIKKQGRHFVLVHGACHGGWCWYKLKPLLEVAGHKVTTLDLAASGIDLRKIEQLHTFH      60


MES4hap1        DYTLPLMELMESLPQRGESHTSWT------------------------------------      84
MES4hap3        DYTLPLMELMESLPQRGESHTSWT------------------------------------      84
MES4hap8        ------MELMESLPQEEKVILVGHSLGGMNLGLVMEKYPQKIYVVVFLAAFMPDSIHSSS      54
MES4hap2        DYTLPLMELMESLPQEEKVILVGHSLGGMNLGLVMEKYPQKIYVAVFLAAFMPDSIHLN-      119
MES4hap4        DYTLPLMELMESLPQEEKVILVGHSLGGMNLGLVMEKYPQKIYVAVFLAAFMPDSIHLN-      119
MES4hap5        DYTLPLMELMESLPQEEKVILVGHSLGGMNLGLVMEKYPQKIYVAVFLAAFMPDSIHSSS      120
MES4hap6        DYTLPLMELMESLPQEEKVILVGHSLGGMNLGLVMEKYPQKIYVAVFLAAFMPDSIHSSS      120
MES4hap7        DYTLPLMELMESLPQEEKVILVGHSLGGMNLGLVMEKYPQKIYVAVFLAAFMPDSIHSSS      120
                      *********. :

MES4hap1        ------------------------------------------------------------      84
MES4hap3        ------------------------------------------------------------      84
MES4hap8        YVLDQGQAK---------------------------------------------------      63
MES4hap2        ------------------------------------------------------------      119
MES4hap4        ------------------------------------------------------------      119
MES4hap5        YVLDQ--------------YFERMQTMNWLDTQFVSYGSPEEPLPSIFFGPKFLAYNLYQ      166
MES4hap6        YVLDQVLFINPILFVIFVKYFERMQTMNWLDTQFVSYGSHEEPLPSIFFGPKFLAYNLYQ      180
MES4hap7        YVLDQVLFINPILFVIFVKYFERMQTMNWLDTQFVSYGSHEEPLPSIFFGPKFLAYNLYQ      180


MES4hap1        ------------------------------------------------------------      84
MES4hap3        ------------------------------------------------------------      84
MES4hap8        ------------------------------------------------------------      63
MES4hap2        ------------------------------------------------------------      119
MES4hap4        ------------------------------------------------------------      119
MES4hap5        LCPPEDVALVSSLGRASSLFLEDLSKSKYLTDEGYGSVKKVYIVCTDDKLLPKEFQKWQI      226
MES4hap6        LCPPEDVALVSSLGRASSLFLEDLSKSKYLTDEGYGSVKKVYIVCTDDKLLPKEFQKWQI      240
MES4hap7        LCPPEDVALVSSLGRASSLFLEDLSKSKYLTDEGYGSVKKVYIVCTDDKLLPKEFQKWQI      240


MES4hap1        ---------------------------------------   84
MES4hap3        ---------------------------------------   84
MES4hap8        ---------------------------------------   63
MES4hap2        ---------------------------------------   119
MES4hap4        ---------------------------------------   119
MES4hap5        DNINSIIETKEIEGADHMAMLSMPKKLCDTLLEIADKYN   265
MES4hap6        DNINSIIETKEIEGADHMAMLSMPKKLCDTLLEIADKYN   279
MES4hap7        DNINSIIETKEIEGADHMAMLSMPKKLCDTLLEIADKYN   279
```
