# Supplementary material for: Structural variation underlies functional diversity at methyl salicylate loci in tomato
Source: PLoS Genet. 2023 May 4;19(5):e1010751. doi: 10.1371/journal.pgen.1010751 (PMC10187894; doi:10.1371/journal.pgen.1010751)
Supplement: S3 Fig — The gene model below is a screenshot from SL4.0 genome version of the exact region which is the insertion showing a transposon insertion between SlMES2 and SlMES3. (PDF) [file pgen.1010751.s003.pdf]

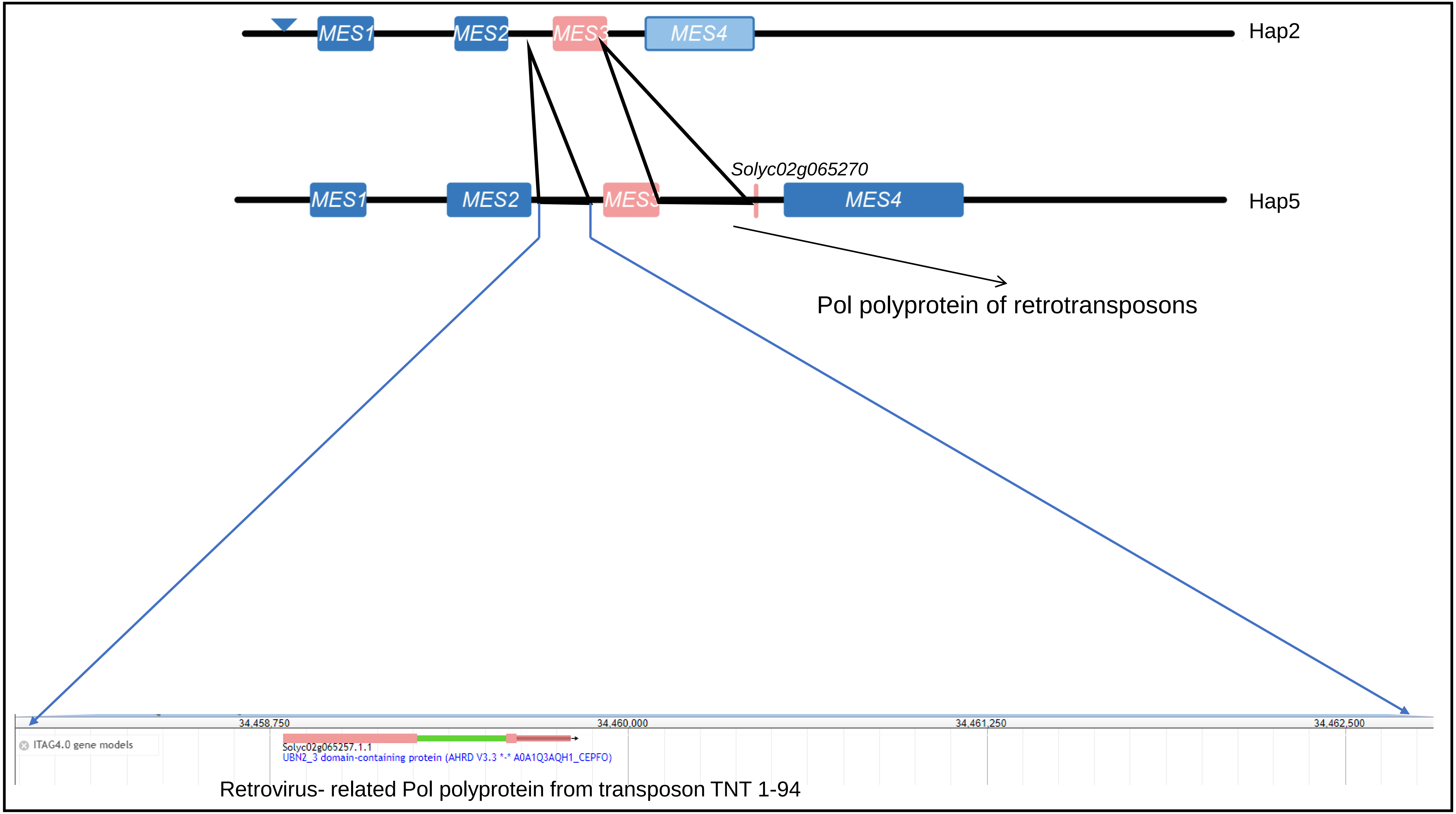

MES1
MES2
MES3
MES4
Hap2
Solyc02g065270
MES1
MES2
MES3
MES4
Hap5
Pol polyprotein of retrotransposons
34,458,750
34,460,000
34,461,250
34,462,500
ITAG4.0 gene models
Solyc02g065257.1.1
UBN2_3 domain-containing protein (AHRD V3.3 *-* A0A1Q3AQH1_CEPFO)
Retrovirus- related Pol polyprotein from transposon TNT 1-94
