# Supplementary material for: Structural variation underlies functional diversity at methyl salicylate loci in tomato
Source: PLoS Genet. 2023 May 4;19(5):e1010751. doi: 10.1371/journal.pgen.1010751 (PMC10187894; doi:10.1371/journal.pgen.1010751)
Supplement: Attachment — Submitted filename: Answers to reviewers comments.docx [file pgen.1010751.s017.docx]

Dear Nathan,

Thank you and the reviewers for the handling of the manuscript on the genetic characterization of loci underlying methyl salicylate levels in tomato fruits. Below, we cover point by point the reviewer’s concerns.

**Reviewer #1:**

A manuscript entitled “Structural variation underlies functional diversity at methyl salicylate loci in tomato” investigates how the allelic variation of four genetic loci shape the quantitative variation of methyl salicylate in tomato fruit and how this variation might have evolved during evolution and domestication. The manuscript contains a large amount of novel interesting information, that can prove useful for both fundamental understanding of metabolic variation in tomato fruit as well as for applied proposes, such as breeding for specific chemical composition of tomato fruit. The quality of the study and the presentation of the results is high, but there are a few points that I’d like to see addressed.

Line 80 “While the levels of methyl salicylate are generally low in red-fruited tomato including heirlooms, some produce intermediate levels of the volatile in ripe fruit, suggesting that selections for yield and disease resistance were at the expense of better flavor, a common concern among consumers” – selection for yield and resistance is indeed often blamed for the poor flavor of many modern tomato cultivars. However, I don’t immediately see how varying levels of MeSa suggest that “selections for yield and disease resistance were at the expense of better flavor”. The data present in literature suggests that the ratio between relatively high and low MeSA tomatoes is roughly 50/50 and fruit type independent. Wouldn’t this suggest that the MeSA fruit phenotype is rather neutral for both flavor and tomato plant well-being? Besides, a lower MeSA content would be rather beneficial since a higher content of this volatile often correlates with off-flavor.

Answ: Thank you for pointing this out. We changed the paragraph accordingly: “Tomato taste panels routinely associate this volatile with low liking as it features the characteristic odor of wintergreen. However, some varieties with moderate levels of methyl salicylate are rated high for overall liking. This may be the result of concentrations of or interactions with other volatiles resulting in overall good flavor [25, 30].”

Besides that, the importance of MeSA as a selection factor for flavour is questionable. The maximum concentration in the accession collection reported here, which represent a wide genetic diversity, is about 30 ppb whereas the odor threshold reported for MeSA is about 40 ppb (Baldwin et al., 2000, HORTSCIENCE) and this is water meaning that in a fruit matrix it would be even higher. The observation that MeSA has often been associated with off-flavor comes from the correlation and regression analyses, which do not necessarily prove the causal effect. In this particular case the off-flavor could be more likely caused by e.g. guaiacol, which often highly correlates to MeSA (mostly due to NSGT), has a comparable concentration range, but a much lower odor threshold.

Answ: The reviewer brings up a valid point. Since odor thresholds can vary depending on the matrix and interactions with other aroma compounds, the levels found in our collection could still contribute to flavor that is discernable by humans. However, Baldwin et al. also showed that the concentration of methyl salicylate in the varieties they tested was above their odor threshold. Since we use a different method for volatile collection (purge and trap vs. headspace) and quantification, the quantities of methyl salicylate do not compare 1 on 1 with those that were reported by Baldwin et al. We agree that the affects on flavor at the *NSGT1* locus are likely the result of differing guaiacol levels. We have modified the section by adding a sentence in lines 81-82: “This may be the result of concentrations of or interactions with other volatiles resulting in good flavor [25, 30].” We also added a line in the discussion section, lines 505-506 noting the benefits of introgressing functional *NSGT1* haplotypes to lower guaiacol levels.

Of the four loci studied only NSGT and MES seem to explain certain amount of variation in MeSA. Line 332 states that “Overall, we could not show functional association of SAMT1 and UGT5” - Nevertheless there's a significant association of MeSA and these two loci in the GWAS. Does this mean SAMT1 and UGT5 are not the genes underlying the variation in these loci?

Answ: No, not at all. Within the red fruited tomato clade, the GWAS showed significant association with MeSA at the two loci, implying genetic variation at these two loci that contribute to volatile levels. However, we could not demonstrate a likely causal variant at *SAMT1* and *UGT5*, contrary to *MES* and *NSGT1*. Yet, the linkage of a variant with, especially, *SAMT1* is quite convincing that this is the gene at the GWAS QTL. To bridge a significant GWAS variant to causal variant, we performed other analyses. The inclusion of variants 3kb upstream, 1 kb downstream and within the gene body did not identify haplotypes and/or variant(s) that are performing better or worse. Similar results for known genes have been reported previously as well (Pereira et al., 2021). Additionally, it is always possible that another gene near *SAMT1* and *UGT5* underlies the GWAS QTL but that would be exceedingly rare. To clarify the results, we changed the paragraph accordingly.

Besides that, accessions with identical MES/NSGT haplotypes show quite different MeSA contents. For instance, in table S3 accession TS-275 - low MeSA - has exactly the same HPT combination as the top producer TS209. Thus, what % variation these do these two loci actually explain? Does this mean there's something else? And why it doesn’t come out of the GWAS?

Answ: Indeed, accessions with the same *MES* and *NSGT1* haplotypes can show quite different MeSA levels. Variants associated with *MES* control around 28% of variation whereas variants at *NSGT* control around 40% of the MeSA variation from the GWAS analysis. First, environmental impacts (heat, drought, wind, some disease pressure) can dramatically alter MeSA levels. Second, *MES* and *NSGT1* don’t explain all variation in the GWAS panel, such as *SAMT1* and *NSGT1* and other minor QTLs that were found. To clarify, we added this information in the discussion section.

The discussion on the evolutionary development of the loci is very interesting, but, in my opinion, the authors have put too much weight on MeSA as a driving force of selection for these loci. The effect of the NSGT locus is certainly not restricted to MeSA only, but it also glycosylates other compounds. The same might hold for MES that might act on other ester substrates, which, as recent studies showed, are present in fruits of wild tomato species. They can also demethylate some non-volatile secondary metabolites that have not been analysed in the tomato material studied here.

Answ: Thank you for pointing this out. Indeed, *NSGT1* is critical for the glycosylation of other compounds in addition to MeSA but the role of *MES* on other compounds that were not measured or not accumulating in measurable quantities can’t be ruled out either. Since *MES* was identified for its association with a methyl salicylate only, we focused on this volatile. However, *MES* may have activities on other volatiles or non-volatiles.

The picture of both structural alterations and expression levels is kind of scattered throughout the manuscript which makes it difficult to grasp. Can these somehow be summarized (at least for MES and NSGT loci, since the other two don't do much) in e.g. a table or a figure so that a reader could in one glance see what likely to cause a certain MeSA chemotype in different haplotypes, e.g. is that a deleterious mutation in a certain gene(s) or expression or both?

Answ: That is a good idea, thanks for pointing this out. To address the situation, we added a table (Table 1) in the manuscript summarizing the results for the *MES* haplotype. However, structural variation at *NSGT1* has been previously investigated in Alonge et al., 2020 and should not be presented herein.

**Reviewer #2:**

Structural variation underlies functional diversity at methyl salicylate loci in tomato.

This is a nice manuscript that takes advantage of the VARITOME collection that has been genotyped and phenotyped by this consortium of partners with the aim to define the origins and evolution of tomato and eventually use of this knowledge in breeding.

In this MS authors focus their attention in the different loci contributing to one of the VOCs that are produced by tomato fruits and generally associated negatively with good tomato flavor MeSA and the authors speculate about a hypothetical agroecological role it could have in the tomato evolution from the center of origin in South and Meso America. The key genes regulating MeSA and their diversity in the tomato clade has been described in a few seminal papers that counted in some with the participation of the authors of this paper. Namely, the key role of the MES cluster and the NSGT have been described in detail from evolution to biochemistry . (MES in Plant Physiology, Volume 191, 110–124; https://doi.org/10.1093/plphys/kiac509 ). NSGT molecular genetics also in Alonge et al (2020) Cell. 2020 Jul 9; 182(1): 145–161.e23. doi: 10.1016/j.cell.2020.05.021

Thus some results seem to me they lack novelty (ie GWAS figures in the PP paper and in the current MS are basi1cally the same) and some of the RT PCR results don’t reveal anything substantially new that was not revealed in the PP paper.

Answ: In terms of the molecular and biochemical nature of *MES*, indeed, this manuscript does not offer substantially more insights. However, this study focuses exclusively on the underlying genetic architecture of MeSA levels in tomato fruits, the identification and functional characterization of 9 MES haplotypes (versus 2) and haplotypes at two minor loci, the extensive structural variations that shapes genetic and phenotypic diversity within a short evolutionary timespan, and the selection of certain haplotypes of *MES* and *NSGT1* at different times and in distinct localities; all of that is novel. The strong interaction of their haplotypes is novel as well and hugely helpful for plant breeders to select the desired combination of *MES* and *NSGT1* in tomato improvement. Regarding the GWAS performed for this manuscript, we included SVs and INDELs in addition to SNP which was published in Frick et al. 2023 paper which is indicated in the methods.

Also de authors describe in the text many details that can be directly obtained from figures or unnecessary. Authors should cleary indicate what data were generated and original for this ms ad which already reported and indicate were to find the original data as required by PlosGenetics.

Answ: The originality of the results is addressed in the previous comment. Additionally, we mention now that several accessions of the 37 accessions used for qPCR were previously shown in Frick et al., 2023.

To discuss about the “functional” diversity in relation to a putative eco/evolutionary advantage is highly speculative as it is based on single measurements at the ripe stage of fruit (from probably/growing under standard in greenhouse conditions) and the role of MeSA in ripe fuir in relation to these aspects has still to be substantiated with more results. MeSA is known to respond to evironmental cues and the authors know and reported in the PP. This is MeSA-centric ripe fruit-centric paper and when talking about interaction with biotic and abiotic conditions, it misses to include in the analysis SA (the well non mediator if these type of interactions, and the genes involved in the Biosynthesis and metabolism (including SA glycosylation and also misses what happens at the vegetative/plant level to address the question of any evolutionary/ecological role. Highly speculative: all in this paper is derived from (single) measurements of MeSA in ripe fruit samples so it should not sound like these fruit MeSA related loci can explain ecological adaptations that may depend on plant SA levels for instance more than in red fruit MeSA.

Answ: The reviewer is correct that having information of SA levels in tomato fruits from the Varitome collection would have been helpful in this specific context (MeSA levels in tomato fruits). However, our study focuses on fruit volatiles, and SA would need to be differently extracted and derivatized prior to GC analysis. To clarify the reviewer’s concern about data collection, the volatile data were obtained from fruits that were collected in multiple harvests (up to 5) from multiple plants grown in the field in Live Oak in FL in 2016. The volatile samples were freshly collected, within a day of harvest. A further analysis of MeSA levels in roots and leaves would have been nice to include but it is outside the scope of this study because this study focuses on ripe fruits. We postulate that high MeSA levels in the fruit are possibly linked to ecological necessity because that is what the data suggest. The haplotype selections are even more clearly showing that both loci underwent strong selection during domestication. Of course, ecological adaptation depends on more than volatiles emitted by ripe fruits. Again, those analyses are beyond the scope of this study. We’d welcome another explanation for the high levels of MeSA in fruits from Ecuador, the association of the non-functional haplotypes at *MES* and *NSGT1*, if an ecological explanation is not valid with the analyses conducted for this study.

Authors take for granted that the triglycosilated form of MeJA produced by NSGT is an end irreversible product and this appears to be the case in tomato but is it possible that some accessions of SP or SLC may have a specific glycosidases that could make this last step reversible by cleaving the triglyco20sid form of MeSA?

Answ: Thank you for the comment. It is indeed plausible that some accessions might have specific glycosidases that could make the last triglycoside conjugation step reversible. However, such glycosidases were not identified from the GWAS panel.

Please give details on how VOCs were determined. If is by cutting the fruits and flow and trap from tomato wedges as in the PP papers is it possible that MeSA production changes during wounding, release and capture and the expression of corresponding biosynthetic and metabolism genes. If so then could it be that the MES steady state levels obtained by RNA extraction of ripe fruit are missing a possible induction produced in the wounded tomato wedges during MeSA capture and also does not capture the eventual activataion of the MES, NSPGT etc in response to the different environmental conditions that the accessions were experiencing during their evolution.

Answ: Thank you for the comment. The red ripe fruits were cut and processed similarly as in the PP paper. The samples were loaded in glass tubes and the air was passed over samples for an hour to collect the volatiles on a SuperQ Resin. Volatiles were then eluted off the column with methylene chloride and ran on GC-MS for analysis. This information is added in the methods. It is certainly possible that MeSA levels increase upon wounding during collection. However, volatiles from all samples were collected similarly, and therefore any differences should be proportional to the amounts in unwounded fruits. More importantly, we believe that our method better simulates what happens when humans slice and eat tomatoes; therefore, the collection of the volatiles appears to be more consistent with the flavor ratings of the fruit in taste panels.
